# The impact of the COVID-19 pandemic on contraceptive methods, abortion, and unintended pregnancy: a cross-sectional study

**DOI:** 10.1186/s12905-023-02512-y

**Published:** 2023-07-04

**Authors:** Fatemeh Rezaei, Leila Amiri-Farahani, Shima Haghani, Sally Pezaro, Fereshteh Behmanesh

**Affiliations:** 1 MSc in Midwifery, Department of Reproductive Health and Midwifery, School of Nursing and Midwifery, Iran University of Medical Sciences, Tehran, Iran; 2 Department of Reproductive Health and Midwifery, Nursing and Midwifery Care Research Center, School of Nursing and Midwifery, Iran University of Medical Sciences, Tehran, Iran; 3 Department of Biostatistics, Nursing and Midwifery Care Research Center, Iran University of Medical Sciences, Tehran, Iran; 4 PhD in Midwifery, The Research Centre for Healthcare and Communities, Coventry University, Coventry, UK; 5 The University of Notre Dame, Fremantle, Australia; 6 Social Determinants of Health Research Center, Health Research Institute, Babol University of Medical Sciences, Babol, Iran

**Keywords:** Contraceptive methods, Unintended pregnancy, Abortion, COVID-19 pandemic, Reproductive behaviors

## Abstract

**Background and Aim:**

By creating an international emergency, the COVID-19 pandemic may have led to compromised reproductive health care, including family planning services, and thus increased unintended pregnancies and unsafe abortions. This study was conducted to compare methods of contraception, abortion, and unintended pregnancies in those served by the health centers of Babol city in Iran, both before and during the COVID-19 pandemic.

**Methods:**

A cross-sectional study was conducted including 425 participants registered to the health centers of Babol city, Mazandaran province, Iran. Using a multi-stage method, 6 urban health centers and 10 rural centers were selected for inclusion. Proportional allocation method was used for sampling those who met the inclusion criteria. A questionnaire was used to collect data in relation to individual characteristics and reproductive behaviors via 6 questions focused upon methods and preparation of contraception, number and type of abortions, and number and causes of unintended pregnancy from July to November 2021. The data were analyzed using SPSS software version 26. Significance level was considered to be p < 0.05 in all tests.

**Results:**

Most participants aged between 20 and 29 years old had a diploma level of education, were housewives and lived in the city. Prior to the pandemic, 32.0% used modern contraceptive methods and 31.6% used these during the pandemic. No change in the combination of contraceptive methods used was observed between these two periods. Approximately two-thirds used the withdrawal method in both periods. The majority of participants in both periods purchased their contraceptives from a pharmacy. Unintended pregnancy increased from 20.4% prior to the pandemic to 25.4% during the pandemic. Abortions increased from 19.1% prior to the pandemic to 20.9% during the pandemic, although these findings were not found to be statistically significant. Contraceptive methods had a statistically significant relationship with age, education, spouse’s education, spouse’s occupation, and place of residence. The number of unintended pregnancies had a significant relationship with age, the educational level of both participants and their spouses and socio-economic status, and the number of abortions had a statistically significant relationship with the age and education level of the spouse (p > 0.05).

**Conclusion:**

Despite there being no change in contraceptive methods compared to the pre-pandemic period, an increase in the number of unintended pregnancies, abortions and illegal abortions was observed. This may be indicative of an unmet need for family planning services during the COVID-19 pandemic.

**Supplementary Information:**

The online version contains supplementary material available at 10.1186/s12905-023-02512-y.

## Introduction

The sudden appearance of the COVID-19 virus and the resulting pandemic put the world into a health crisis. This was particularly concerning for cisgender women and girls, who are disproportionately affected in emergency situations and face multiple sexual and reproductive health challenges [1].

Reproductive behavior as a human phenomenon occurs in the context of cultural beliefs and socio-economic status. During the COVID-19 crisis in particular, loss of work and health insurance reduced access to health care for many people [2]. This in turn may have affected reproductive behaviors. These behaviors relate to reproduction [3], (e.g., number of children, spacing between children, use of contraceptives, unintended pregnancy, abortion, and reproductive intention).

Various factors such as age, family income, ethnicity, education [4, 5], religion [6, 7], marriage age, satisfaction with marital life [8, 9], number of living children [4, 9], women’s employment [4], duration of marriage, attitude about the use of contraceptives [8], ideal number of children, place of residence, and exposure to mass media [6] influence reproductive behaviors. Crises such as natural disasters (including floods, earthquakes, storms, etc.) and diseases can also affect reproductive behaviors, disrupt the availability of contraceptive methods and services and evoke short-term decreases in fertility [10, 11]. Nevertheless, these factors are yet to be understood in relation to the COVID-19 pandemic.

After the Ebola outbreak in Sierra Leone, a decrease in the receipt of family planning services was observed [12]. Furthermore, in Guinea, the number of family planning visits increased by 47% during the first three months of the Ebola outbreak, and then at the peak of the Ebola outbreak, there was a 51% drop in visits compared to pre-Ebola levels [13]. Moreover, during the Zika outbreak, when quality contraceptive services became available, the use of contraceptives to prevent unintended pregnancy and adverse birth outcomes reportedly increased [14]. Similar impacts may have occurred due to the COVID-19 pandemic.

By disrupting the supply chain of contraceptives, the COVID-19 pandemic led to restrictions on the production, distribution, and availability of contraceptives [15]. Moreover, health service providers were redirected from providing family planning services to responding directly to clinical cases of COVID-19 [16–18]. Many were also unable to attend health centers due to quarantine or fear of exposure to COVID-19 [19]. Such changes in the use of contraceptives are concerning as they can lead to unintended pregnancy, unsafe abortion, and consequently, perinatal mortality [20].

Globally, the COVID-19 pandemic has affected family planning in a variety of ways. For example, in Nigeria, the prevalence of using modern contraceptive methods increased from 30.1% before the COVID-19 pandemic to 35.1% during quarantine [21]. In Germany, the reported demand for condoms also increased sharply [22]. Nevertheless, some evidence suggests there was a decrease in the use of contraceptive methods. For example, In South Africa evidence suggests that between February and April 2020, the Couple-Years of Protection (CYP) decreased by almost 50% compared to the previous two years [23]. In this same study, the use of oral contraceptive pills was evidenced to have increased, and the use of injectable hormonal contraceptives, hormonal implants, and IUD use reportedly decreased during the post-quarantine period, compared to the average intake in the previous two years. The number of female sterilization performed monthly, including during the quarantine period, remained constant [23]. In the United States of America, the attitudes of many have changed toward contraception due to the COVID-19 crisis, whereby many (23%) turned to more effective and longer-term contraceptive methods [24]. Nevertheless, approximately 33% also canceled or delayed the contraceptive visit [24] Such inconsistencies in the evidence base call for further research.

When the need for family planning services are not met, the number of unintended pregnancies increases [25]. Quarantines can also increase rates of intimate partner violence, including coercion and sexual assault, leading to further unplanned pregnancies [26], and thus increased rates of abortions [26]. A lack of access to abortion services also leads to the reduction of safe abortion services provided in health centers, and needless mortality. The global evidence comparing abortion both during and prior to the COVID-19 pandemic is not clear. In South Africa, abortion services provided during the COVID-19 period decreased overall by 5%, whilst the number of abortions performed in the first trimester increased by 2% and the number of abortions performed in the second trimester of pregnancy decreased by 17% [23]. In the West African country of Burkina Faso, the history of spontaneous abortion or stillbirth reported by women increased by 41% after 12 months of the epidemic compared to the pre-epidemic period [27]. Yet in relation to the impact of COVID-19 on first trimester spontaneous abortion, Cosma et al. (2020) demonstrated that contracting COVID-19 did not lead to spontaneous abortion in the first trimester [28]. In France, the total frequency of abortion (including medical, surgical and home abortion) decreased by 3.5% between 2019 and 2020, and yet the frequency of medical abortions during the pandemic increased in 2020 compared to 2019 [29]. It will be important for future research to expand global understandings in relation to abortion during and prior to the COVID-19 pandemic in order to both prepare for future pandemics and plan adequately for future populations.

Globally, the COVID-19 pandemic has had evidential and contradictory effects on pregnancy prevention. Though some studies report an increase in the use of modern contraceptive methods, others report a decrease in the use of modern methods. Evidence is contradictory in many cases, and comparisons of the frequency of spontaneous abortion and the cause of medical abortion (therapeutic or intentional) recorded prior to, and during the COVID-19 pandemic remain under investigated. Considering the illegality and lack of provision of abortion services without medical indication in Iran, data in relation to abortion and its types in Iran are particularly limited. Considering the above, the present study aimed to compare [1] methods of contraception and the place of its preparation, [2] abortion and its types and [3] unintended pregnancy and the cause of unintended pregnancy in women attending the health centers in Babol city, Iran, both before and during the COVID-19 pandemic.

## Methods

### Study design

The current cross-sectional study included women (presumed cisgender) of reproductive age and registered with urban and rural health centers in Babol city, Mazandaran province, Iran. Babol city is the most populated city in the Mazandaran province and is the second most populous city in northern Iran. The total number of people covered by comprehensive health centers in Babol city is 505,679, and the number of married women of reproductive age covered is 96,095. Married women of reproductive age in Babol city comprise 22.10% of the total population. The population of married women of reproductive age in the whole country is 21.23%. According to the Iranian Statistical Center, the total fertility rate in Babol city in 2018 was 1.2, which is lower than the total fertility rate in the country overall (1.8) [30]. In total, Babol city has 27 comprehensive rural health centers and 13 comprehensive urban health centers (Additional file 1).

### Study sample

To determine the minimum sample size necessary to estimate the frequency of abortion (with the lowest prevalence among the variables to calculate the highest sample size), at a confidence level of 95% and with the estimation accuracy of d = 0.04, after quantification in the following formula, a minimum sample size of 415 was estimated to be required.

$$n=\frac{{\varvec{z}}_{1-\raisebox{1ex}{$\propto $}\!\left/ \!\raisebox{-1ex}{$2$}\right.}^{2} \varvec{p}\varvec{q}}{{\varvec{d}}^{2}}=\frac{{1.96}^{2}\times 0.78\times 0.22}{{0.04}^{2}}=412\approx 415$$

Sampling was multi-staged. Firstly, stratified sampling was used to delineate each of the six districts of Babol city. Subsequently, 2–3 rural health service centers and 1–3 urban health service centers were selected randomly from each class. Subsequently, all electronic patient codes which were a multiple of 15 were selected at random. If the coded patient met the inclusion criteria for the study, they were invited to participate. Sampling from each center continued until a sample size of 425 was reached between August and November 2021. Participants met the study’s inclusion criteria if they were women (presumed cisgender) aged 15–49 married for at least 2 years and currently co-habiting with a spouse, absent of primary and secondary infertility, absent of conditions which interfere with fertility, and absent of severe mental disorders (known mental disorders diagnosed by a physician or/and requiring medication). Eligible participants were also required to have an active electronic file in the Sib system and willing to participate when invited via telephone call. In total, the electronic files of 802 patients defined as being of reproductive age by the World Health Organization (WHO) (15–49 years old) were reviewed [31], of which 377 were excluded as they either did not meet the study’s inclusion criteria or declined participation.

### Outcome measures and measurements

The data collection tool included a personal profile questionnaire and reproductive behavior questionnaire. The personal profile questionnaire included items relating to: age, education, spouse’s education, occupation, spouse’s occupation, place of residence, and socioeconomic status. Contraceptive methods and their place of preparation, number and type of abortion, number of unintended pregnancies and the cause of unintended pregnancy were also investigated using the reproductive behavior questionnaire. This questionnaire was designed by Behmanesh et al. in 2014 after which time; its validity and reliability were approved [32]. This questionnaire contains 20 questions and measures items such as the total number of pregnancies, and information about current pregnancy, abortion, fertility intentions, unintended pregnancy, causes of unintended pregnancy, length of time between marriage and first pregnancy, number of children, number of neonatal mortalities and the length of time between each child being born. In the current study, this questionnaire was adapted and subsequently sent to 9 experts for review. Content and face validity were subsequently quantitatively and qualitatively examined and confirmed. Since the questions were completely independent from each other, they were not considered in need of being checked for reliability.

Questionnaires were completed by the research team where appropriate, using the electronic file data within the Sib system collected one year before the COVID-19 pandemic and once again (July to November 2021) after the commencement of Iran’s vaccination program roll out. Absent data which could not be collected via the Sib system (e.g., type of abortion, unintended pregnancy and the cause of unintended pregnancy in the period before and during the COVID-19 pandemic) was collected via telephone.

### Data analysis

Data were analyzed after coding was completed using SPSS software version 26. Descriptive statistics were used to analyze individual characteristics, methods of contraception and where provided, abortion and its types, unintended pregnancy and the cause of unintended pregnancy (including absolute and relative frequency, mean and standard deviation) to compare them in the period before and during the COVID-19 pandemic. To identify any correlation between individual characteristics, contraceptive methods and its place of preparation, abortion and its types, unintended pregnancy and the cause of unintended pregnancy, chi-square and Fisher tests were used. The level of significance in all tests was considered to be p < 0.05.

## Results

The highest frequencies of participants (36.5%) were aged between 20 and 29 years. The highest frequency of education level (41.4%) was diploma level education. During the COVID-19 pandemic, 79.1% of participants were reportedly housewives. Among spouses, the highest frequency of education related to university education level (34.4%) and below diploma level education (34.8%). The majority of spouses (80.7%) were self-employed, and over half of participants (60%) lived in the city and had relatively favorable socio-economic status (67.1%) (Table 1).

**Table 1:** Frequency distribution of participants and their spouses’ individual characteristics during COVID- 19 pandemic

Individual characteristics		N	%
**Age (years)**	**< 20**	22	5.2
	**20–29**	155	36.5
	**30–39**	151	35.5
	**40–49**	97	22.8
**Education**	**< Diploma**	114	26.8
	**Diploma**	176	41.4
	**University education**	135	31.8
**Occupation**	**Housewife**	336	79.1
	**Employed**	89	20.9
**Spouses’ education**	**<Diploma**	148	34.8
	**Diploma**	131	30.8
	**University education**	146	34.4
**Spouses’ occupation**	**Employee**	82	19.3
	**Selfemployed**	343	80.7
**Residence**	**Urban**	255	60.0
	**Rural**	170	40.0
**Socio-economic status**	**Unfavorable**	71	16.7
	**Relatively favorable**	285	67.1
	**Favorable**	69	16.2

The most common method of contraception used by participants in both periods was the withdrawal method. Usage of this method increased from 57.9% before the pandemic to 58.6% during the pandemic. The frequency of using modern contraceptive methods decreased from 32.0% in the period before the COVID-19 pandemic to 31.6% during the COVID-19 pandemic. Yet no statistically significant change was observed between the two periods. In the period before the COVID-19 pandemic, 86.4% of participants who used contraceptives obtained these from a pharmacy. This frequency increased to 93.5% during the pandemic, although this difference was not statistically significant (Table 2).

**Table 2:** Comparison of contraceptive methods and where they were obtained from before and during COVID- 19 pandemic

Variables		During the COVID-19 pandemic		Before the COVID-19 pandemic		p-Value
		N	%	N	%	
**Contraceptive methods**	**Unused**	43	10.1	42	9.8	*p = 0.375
	**Surgical methods**	26	6.1	27	6.4	
	**IUD**	3	0.7	3	0.7	
	**Hormonal methods**	17	4.0	19	4.5	
	**Condom**	90	21.2	85	20.0	
	**Withdrawal**	246	57.9	249	58.6	
**Place contraceptive methods obtained**	**Health center**	15	13.6	7	6.5	*p = 0.083
	**Pharmacy**	95	86.4	100	93.5	

*Chi-square test, Significance level: p < 0.05, bold entries are significant results and are related to statistical test

With regards to the relationship between personal characteristics and contraceptive methods, contraceptive methods had a statistically significant relationship with age, education, spouse’s education, spouse’s occupation, and place of residence. With increasing age, the frequency of using modern methods of contraception increased, and were used predominantly by those aged between 40 and 49 years old (43.3%). The frequency of using modern methods of contraception was higher in participants and those with university-educated spouses. The frequency of using modern methods of contraception in those who spouse was an employee was more than that of those who spouses were self-employed. The frequency of using modern contraceptive methods was higher in participants living in urban areas than in those living in rural areas. The frequency of using modern methods of contraception increased with the advancement of socio-economic status. The method of contraception used was not related to occupation (Table 3).

**Table 3:** The relationship between individual characteristics and contraceptive methods and unintended pregnancy during COVID-19 pandemic

Individual characteristics		Contraceptive methods												P value	Unintended pregnancy						P value		
		Unused		Surgical methods		IUD		Hormonal methods		Condom		Withdrawal			0		1		≥ 2				
		n	%	N	%	n	%	n	%	n	%	N	%		n	%	n	%	n	%			
**Age (year)**	**< 20**	3	13.6	0	0	0	0	0	0	5	22.8	14	63.6	*p < 0.001	21	95.5	1	4.5	0	0	*p = 0.03		
	**20–29**	20	12.9	2	1.3	1	0.6	8	5.2	26	16.8	98	63.2		124	80	28	18.1	3	1.9			
	**30–39**	15	9.9	4	2.6	0	0	8	5.3	38	25.2	86	57		104	68.9	37	24.5	10	6.6			
	**40–49**	4	4.1	21	21.6	2	2.1	3	3.1	16	16.5	51	52.6		68	70.1	22	22.7	7	7.2			
**Education**	**< Diploma**	5	4.4	14	12.3	0	0	6	5.3	17	14.9	72	63.2	*p < 0.001	76	66.7	133	75.6	108	80	*p = 0.009		
	**Diploma**	19	10.8	9	5.1	3	1.7	11	6.3	25	14.2	109	61.9		28	24.6	33	18.8	27	20			
	**University education**	18	13.3	4	3.0	0	0	2	1.5	43	31.9	68	50.5		10	8.8	10	5.7	0	0			
**Spouses’ education**	**< Diploma**	12	8.1	13	8.8	0	0	9	6.1	23	15.5	91	61.5	*p < 0.001	98	66.2	38	25.7	12	8.1	*p = 0.01		
	**Diploma**	11	8.4	8	6.1	3	2.3	8	6.1	17	13.0	84	64.1		101	77.1	24	18.3	6	4.6			
	**University education**	19	13	6	4.1	0	0	2	1.4	45	30.8	74	50.7		118	80.8	26	17.8	2	1.4			
**Occupation**	**Housewife**	31	9.2	22	6.5	3	0.9	19	5.8	65	19.3	196	58.3	**p = 0.16	253	75.3	70	20.8	13	3.9	*p = 0.28		
	**Employed**	11	12.4	5	5.6	0	0	0	0	20	22.5	53	59.5		64	71.9	18	20.2	7	7.9			
**Spouses’ occupation**	**Employee**	6	7.3	6	7.3	0	0	2	2.4	28	34.1	40	48.8	*p = 0.01	65	79.3	16	19.5	1	1.2	*p = 0.22		
	**Selfemployed**	36	10.5	21	6.1	3	0.9	17	5	57	16.6	209	60.9		252	73.5	72	21	19	5.5			
**Residence**	**Urban**	27	10.6	23	9.0	2	0.8	10	3.9	56	22.0	137	53.7	*p = 0.04	190	74.5	54	21.2	11	4.3	*p = 0.87		
	**Rural**	15	8.8	4	2.4	1	0.6	9	5.3	29	17.1	112	65.8		127	74.7	34	20	9	5.3			
**Socio-economic status**	**Unfavorable**	4	5.6	5	7.0	1	1.5	5	7.0	7	9.9	49	69.0	**p = 0.02	48	67.6	15	21.1	8	11.3	*p = 0.02		
	**Relatively favorable**	24	8.4	17	6	1	0.4	13	4.5	64	22.5	166	58.2		214	75.1	63	22.1	8	2.8			
	**Favorable**	14	20.3	5	7.2	1	1.4	1	1.4	14	20.3	34	49.4		55	79.7	10	14.5	4	5.8			

*Chi-square test, **Fisher Test, Significance level: p < 0.05, bold entries are significant results and are related to statistical test

Approximately one fifth (20.4%) of participants reported a history of unintended pregnancy prior to the pandemic. This frequency increased to 25.4% during the pandemic. Inappropriate timing (e.g., wanting to become pregnant at a different time) was reported as the most common cause of unintended pregnancy both before (48.3%) and during the COVID-19 pandemic (39.9%) (Table 4).

**Table 4:** Comparison of number and type of abortion with number and reasons why pregnancy was unintended before and during COVID- 19 pandemic

Reproductive behavior		Before the COVID-19 pandemic		During the COVID-19 pandemic		P-Value
		n	%	n	%	
**Unintended Pregnancy**	0	338	79.6	317	74.6	*p = 0.215
	1	69	16.2	88	20.7	
	**≥** 2	18	4.2	20	4.7	
**Reasons why pregnancy was** **unintended**	Worries about economic difficulties	15	17.3	21	19.5	^**^p = 0.52
	Disease	5	5.7	5	4.6	
	Spacing of children	15	17.3	20	18.4	
	Wrong timing	42	48.3	43	39.9	
	Fear of COVID-19	0	0	4	3.7	
	Sufficient number of children reached	10	11.4	15	13.9	
**Abortion number**	0	344	80.9	336	79.1	*p = 0.751
	1	63	14.9	71	16.7	
	≥ 2	18	4.2	18	4.2	
**Abortion type**	Spontaneous	52	64.2	55	61.8	*p = 0.949
	Induced	18	22.2	21	23.6	
	Illegal	11	13.6	13	14.6	

*Chi-square test, **Fisher Test, Significance level: p < 0.05, bold entries are significant results and are related to statistical test

Regarding the relationship between individual characteristics and unintended pregnancy, our results demonstrate that the number of unintended pregnancies had a significant relationship with age, the educational level of both participants and their spouses and socio-economic status (P < 0.05). The number of unintended pregnancies increased with age. The highest level of abortion history (29.9%) was observed in participants aged 40–49 years. The number of unintended pregnancies had an inverse relationship with the level of education in that with the increase in the education levels of both participants and their spouses, the number of unintended pregnancies decreased. The lowest frequency found in relation to this was in those with a level of university education (p = 0.001). The number of unintended pregnancies was also inversely related to socio-economic status and was highest in that those with an unfavorable socio-economic status (32.4%). The number of unintended pregnancies had no significant relationship with other individual characteristics (Table 3).

Abortion rates increased from 19.1 to 20.9% during the pandemic. The highest frequency in the type of abortion in both periods before (64.2%) and during the COVID-19 pandemic (61.8%) was spontaneous abortion (Table 4). Regarding the relationship between individual characteristics and the number and type of abortion, results demonstrated that the number of abortions had a statistically significant relationship with the age and education level of the spouse (P < 0.05). With increasing age, the number of abortions increased. The highest number of abortions (27.8%) occurred in those aged between 40 and 49 years. Also, abortion was 30.4% higher among those whose spouses had an educational below diploma level. The number of abortions had no statistically significant relationship with other individual characteristics. There was also no statistically significant relationship between the type of abortion and any of the variables of individual characteristics (Table 5).

**Table 5:** Relationship between individual characteristics and number of abortions and type of participant before and during COVID- 19 pandemic

Individual characteristics		Abortion type						p-Value	Abortion type						p-Value
		0		1		≥ 2			Spontaneous		induced		illegal		
		N	%	n	%	n	%		N	%	n	%	n	%	
**Age(years)**	**< 20**	19	86.4	3	13.6	0	0	**p = 0.03	2	66.7	1	33.3	0	0	**p = 0.05
	**20–29**	131	84.5	22	14.2	2	1.3		11	45.8	10	41.7	3	12.5	
	**30–39**	116	76.8	29	19.2	6	4.0		24	68.6	8	22.8	3	8.6	
	**40–49**	70	72.2	17	17.5	10	10.3		18	66.7	2	7.4	7	25.9	
**level of education**	**<Diploma**	91	79.8	18	15.8	5	4.4	*p = 0.80	14	60.9	6	26.1	3	13	*p = 0.77
	**Diploma**	135	76.7	34	19.3	7	4		24	58.5	9	22	8	19.5	
	**University education**	110	81.5	19	14.1	6	4.4		17	68	6	24	2	8	
**Spouses’ level of education**	**<Diploma**	103	69.6	37	25.0	8	5.4	*p = 0.01	29	64.4	10	22.2	6	13.3	**p = 0.59
	**Diploma**	112	85.5	14	10.7	5	3.8		10	52.6	4	21.1	5	26.3	
	**University education**	121	82.9	20	13.7	5	3.4		16	64	7	28	2	8	
**Occupation**	**Housewife**	268	79.8	57	17.0	11	3.3	*p = 0.16	41	60.3	18	26.5	9	13.2	**p = 0.51
	**Employed**	68	76.4	14	15.7	7	7.9		14	66.7	3	14.3	4	19.0	
**Spouses’ Occupation**	**Employee**	68	82.9	9	11.0	5	6.1	*p = 0.22	10	71.4	3	21.4	1	7.2	**p = 0.83
	**Selfemployed**	268	78.1	62	18.1	13	3.8		45	60	18	24	12	16	
**Residence**	**Urban**	207	81.2	36	14.1	12	4.7	*p = 0.19	31	64.6	8	16.6	9	18.8	*p = 0.17
	**Rural**	129	75.9	35	20.6	6	3.5		24	58.5	13	31.7	4	9.8	
**Socio-economic status**	**Unfavorable**	53	74.6	11	15.5	7	9.9	*p = 0.14	11	61.1	4	22.2	3	16.7	**p = 0.99
	**Relatively favorable**	229	80.4	47	16.5	9	3.2		35	62.5	13	23.2	8	14.3	
	**Favorable**	54	78.3	13	18.8	2	2.9		9	60.0	4	26.7	2	13.3	

*Chi-square test, **Fisher Test, Significance level: p < 0.05, bold entries are significant results and are related to statistical test

## Discussion

Our findings report comparisons between contraceptive methods, the place of its preparation, unintended pregnancy, the causes of unintended pregnancy, the number and types of abortion in the period before and during the COVID-19 pandemic and the relationship of individual characteristics with each of these variables.

The frequency of using modern contraceptive methods decreased when compared to the period before the COVID-19 pandemic, and changes in the combination of contraceptive methods were observed, though these were not statistically significant. In the period before and during the COVID-19 pandemic, most participants procured their preferred method of contraception via pharmacies, although this difference was also not statistically significant. Contraceptive methods had a statistically significant relationship with age, the education level of participants and spouses, occupation and place of residence. These findings in particular are consistent with those reported by Emery and Koops (2022), in the Republic of Moldova [33]. These findings are also similar to those presented in African countries, where the economic recession caused by the pandemic was shown to reduce the desire to get pregnant, leading to an increase in the use of modern contraceptives [34]. Due to the Iran’s population growth policies and strict family planning laws, there is a lack of access to modern methods of contraception, and consequently many have no choice but to rely upon the withdrawal method for contraception. The pandemic only exacerbated this problem. Similarly in sub-Saharan Africa, many did not change their contraceptive status during the COVID-19 pandemic [35]. In future it will be important to increase access to the most effective methods of contraception to increase global health overall.

In contrast to our study findings, the frequency of contraceptive use in Burkina Faso increased from 41% in the pre-pandemic period to 50% during the pandemic [27]. This contrast may be explained in part by the fact that contraception is free in Burkina Faso, unlike in Iran. In the United States of America, evidence highlights that many women’s attitudes towards contraception changed due to the COVID-19 pandemic, whereby the use of more effective and long-term contraceptive methods increased [24]. Similarly, in Nigeria the prevalence of using modern contraceptive methods increased from 30.1% before the epidemic to 35.1% during quarantine [21]. In Senegal, the frequency of new users of long-acting methods (IUDs and implants) also increased significantly compared to the pre-pandemic period [36]. Such findings provide useful planning opportunities for future pandemics and increased global population health. In this pursuit, future Iranian policies may usefully seek to acquire free contraception for citizens.

In developed countries, barriers to access to family planning are still reported. For example, in Diamond-Smith et al.‘s (2021) study conducted in the United States of America approximately half of contraceptive users who sought care during the COVID-19 pandemic reported at least one barrier to contraception [37]. Likewise, in Lin et al.‘s (2021) study in the United States of America, 17% reported difficulty accessing contraceptives during the [38]. Barriers to access to contraceptive methods were not investigated in our research. Yet whilst these studies may not be comparable, they highlight that barriers to access to reproductive services exist in a variety of contexts and are not confined to the least developed countries in the world. Global efforts are thus required to improve reproductive healthcare throughout the world.

Our findings demonstrate that with increasing age, the use of modern contraceptive methods also increased. The withdrawal method of contraception was used more frequently by younger participants. Yet with increasing age, the use of this method decreased. These results emulate those reported elsewhere both in Iran [39], Nigeria and Guinea [21, 40]. In contrast, during the COVID-19 pandemic, the frequency of using contraceptive methods was higher in younger participants in Bangladesh [41]. This finding may demonstrate younger people’s desire to prevent unintended pregnancy in favor of continued education and job opportunities [41]. These needs reflect a greater need for access to a wider variety of contraception worldwide.

The frequency of using modern contraceptive methods was higher in those with university education. Moreover, the non-use of contraception in those with university education was more frequent than in those with other education levels. This may be because university education is more common in younger people, who also may desire more pregnancies. Those with a university education were twice as likely to use condoms as a contraceptive method than those with other levels of education. Surgical methods were more common in older participants, particularly before the introduction of strict birth control laws in Iran. Moreover, despite the fact that the withdrawal method was the most commonly used by those of all educational levels, the frequency of using this method decreased with increasing education level, and it was least frequently used by those with a university education. Similarly, elsewhere condom use has been found to be higher in those with a level of higher education than those with a lower level of education [39]. For example, during the COVID-19 pandemic the use of modern contraceptive methods in Nigerians with a level of higher education was 4 times higher than in their counterparts without formal education [21]. Similar results have also been reported elsewhere [35, 42–44]. This may indicate that people with a higher level of education have better knowledge about using contraceptives. Thus, increasing levels of education and access to modern contraception may increase contraceptive usage in general.

The findings of the present study did not show a statistically significant relationship between participants’ occupation and contraceptive methods. This finding is consistent with those reported in Guinea [40], yet not in Iran, where a statistically significant relationship between occupation and contraceptive method has previously been found [39]. Further research in this area with larger sample sizes may usefully provide clarity and future directions in the reproductive health of Iranians.

There was a statistically significant relationship between the occupation of participants’ spouses and the method of contraception. The frequency of condom use for example was higher among those whose spouses were employed rather than self-employed. This may be because the education level of employees is higher in those who are employed rather than self-employed. Indeed, our findings demonstrate that the use of condoms in those whose spouses had a university education was more frequent than in those with other educational levels. The use of the withdrawal method was also more common among those whose spouses were self-employed than among those whose spouses were employees. These findings in particular have been confirmed elsewhere [40]. As an example, in Bangladesh the use of modern contraceptive methods was similarly higher among those whose spouses worked in business or worked in the private sector than among those whose spouses had government jobs [41]. More qualitative research may illuminate the reasons behind such findings in future.

There was a statistically significant relationship between participants’ place of residence and method of contraceptive in that the frequency of using modern contraceptive methods was higher among those living in urban areas. The use of surgical methods and condoms was also more frequent among those living in urban areas. The use of the withdrawal method was more frequent in rural settings. Considering the severe limited access to contraception in rural areas, this finding is not surprising, and has been confirmed elsewhere [21, 41], and previously in Iran [39]. Future family planning services require greater coverage in such areas in pursuit of greater health for all.

We identified a statistically significant relationship between socio-economic status and method of contraception used in that the frequency of using modern contraceptive methods increased with the improvement of socio-economic status. This same finding has been reported from Nigeria [21]. In the present study, the use of condoms increased with higher levels of socio-economic status. Although the withdrawal method was the most common method of contraception used, those with unfavorable socio-economic status had a higher frequency of using this as a method than those with other socio-economic statuses. The use of condoms has been found to be more frequent in those who have a better economic status, while the use of oral contraception has been found to be more frequent in those economically weaker [39]. The cost of condoms is higher in Iran. Therefore, this finding is not surprising as people with a better economic level will ultimately have greater ability to buy condoms.

Little changes were observed in the number of abortions performed compared to the period before the COVID-19 pandemic. The highest frequency in the type of abortion in both periods was spontaneous abortion. The frequency of medical and illegal abortion also increased slightly. In France, the total number of abortions decreased, but the frequency of medical abortions in clinics and medical abortions at home increased [29]. In order to reduce risky sexual behaviors as a result of quarantine [45], unintended pregnancies and consequently, abortions also decreased [46]. In Burkina Faso during the pandemic, numbers of terminations of pregnancy (abortion or stillbirth) increased by 41% during the pandemic compared to the pre-pandemic period [27]. This study did not distinguish between the causes of pregnancy termination (stillbirth, intentional abortion or spontaneous abortion), but the possibility that some of these reasons were caused by the COVID-19 virus during pregnancy cannot be disregarded [47]. Higher rates of intentional abortion, reduced use or access to perinatal care, and reduced quality of care may also explain this increase [48]. Yet regarding the effect of COVID-19 on first trimester spontaneous abortion, contracting COVID-19 has not been found to lead to increases in first trimester spontaneous abortions [28]. Future research may usefully seek to understand these differences retrospectively.

In relation to abortion services provided by abortion clinics, an overall decrease of 5% in abortion services provided in the early months of the COVID-19 pandemic was identified in South Africa [23]. In the state of Louisiana, South America in the first months of the quarantine, the number of monthly abortions in clinics whose services were disrupted decreased by 46% [49]. Whilst the method of conducting these studies is different from the current research, these disruptions to abortion as healthcare are notable and may be applicable worldwide.

Regarding the relationship between individual characteristics and the number and type of abortion undertaken, the results of our study showed that the number of abortions undertaken increased with age. Similar findings have been reported elsewhere [50, 51]. No statistically significant relationship was observed between education level and abortion. Yet this has not been found to be the case elsewhere in Iran, where the highest frequency of intentional abortion was previously found in those with a higher level of education [51]. According to the illegality of abortion in Iran, the frequency of abortion is higher in those with higher income [51]. Future changes in the law may permit safer abortions worldwide.

Abortions were 30.4% higher among those whose spouses had less than a diploma level of education. Elsewhere in Iran, abortion was found to be more common in those whose spouses had a higher level of education [51, 52]. This may be because those with a higher level of education have higher incomes. The frequency of unintended pregnancy during the COVID-19 pandemic was higher in those with lower income. Yet with the improvement of the socio-economic status, the number of unintended pregnancies decreased. This finding has similarly been reported elsewhere [53]. Likewise to results reported elsewhere [50], spouse’s occupation and place of residence had no statistically significant relationship with the number and type of abortion. Participants’ occupation equally had no statistically significant relationship with the number and type of abortion; this finding has been reproduced elsewhere (e.g., in Brazil and Iran) [18, 50, 52]. Yet one study found that intentional abortion in employed participants was twice that of those not employed [51]. This may be due to higher incomes and a desire for career progression. Spontaneous abortions have also been found to be fewer in rural areas of Iran [54], indicating a greater need for healthcare in such areas.

Participants’ socio-economic status had no statistically significant relationship with the number and type of abortion, as has been identified previously [18, 50]. Yet in another study, rates of intentional abortion were found to be higher in those with a higher socioeconomic status, while rates of unintentional abortion were higher where levels of socioeconomic status were lower [55]. Another study conducted in Iran found that 3 out of 5 cases of intentional abortion occurred in those who were in the third and fourth quartile of higher income [51]. Due to the illegality of abortion in Iran, there is little safe abortion available, and only those with high income levels can afford it [51]. Thus, greater accesses to free abortion services are required.

Our results show that numbers of unintended pregnancies increased during the pandemic as they did in the United Kingdom [56], and Burkina Faso [27]. In the present study, inappropriate timing was the most common reason for a pregnancy being unintended in both periods. Worrying about financial issues increased from before the pandemic compared to after the pandemic. During the pandemic, 3.7% of participants cited that the reason for their unintended pregnancy related to a fear of COVID-19 virus. These findings may usefully inform future family planning activities in times of crisis or in the context of future pandemics.

The results of our study showed the number of unintended pregnancies increased with increasing age, as they do in other parts of Iran [53] and elsewhere [57]. Yet in a large study in South Asia adolescents were found to be 1.42 times more likely to have an unintended pregnancy [58]. Older women on the other hand are more likely to plan their pregnancy in other contexts [57]. Further qualitative research may usefully identify the reasons behind such differences.

In our study, the number of unintended pregnancies decreased with increased levels of education. This finding has been replicated in previous studies [40, 53, 59]. This finding in particular may be indicative of a greater awareness on the use of contraceptives. Equally, in the present study, the number of unintended pregnancies decreased where spouses’ level of education was higher. This has also been found to be the case in previous studies [53, 60]. Thus, increased education for all in Iran may further decrease numbers of unintended pregnancies along with subsequent abortions.

As has also been found in Guinea [40], our findings did not show a statistically significant relationship between occupation and unintended pregnancy. Yet in another study, unintended pregnancy was found to be more common in housewives than in employed women [53]. The findings of our research also did not show a statistically significant relationship between the place of residence and unintended pregnancy. Yet findings from South Asia and sub-Saharan Africa show that unintended pregnancy is less frequent in urban [58, 61]. This may be because there is greater access to family planning services in urban areas. Those living in rural areas also have less knowledge in using contraceptive methods and emergency contraception [62]. Thus, greater access to family planning services and education relating to contraception is required throughout Iran.

## Conclusion

The findings presented here provide important insights in relation to the impact of the COVID-19 pandemic on contraceptive methods, abortion, and unintended pregnancy in Iran. Such findings may be useful in planning for future crises including future pandemics and highlight a potential lack of access to family planning services, particularly in rural areas. Increased access to free and safe contraceptive methods and abortions is suggested to reduce the number of unsafe illegal abortions captured here.

## Strengths and weaknesses, suggestions for future research

The use of a standard questionnaire and multi-stage sampling from all 6 districts of Babol city is a key strength of this study. Due to the impossibility of face-to-face sampling during the COVID-19 pandemic, we were limited in using only systematic and telephone sampling methods. Due to the illegality of abortion without indication in Iran, individuals may not have provided a true report of the number of illegal abortions. Another limitation of this study is that it was conducted after the start of the covid-19 vaccination program in Iran. Variables investigated in the present study may have undergone changes after the vaccination program roll out. Replication of this study and further qualitative research in other cities of Iran in future is suggested to further compare findings and enable deeper understandings.

## Electronic supplementary material

Below is the link to the electronic supplementary material.

**Additional File 1**: Frequency distribution of allocation of a proportion of selected urban and rural centers of Babol city

## Data Availability

All the information obtained from this study is not available to the public due to the confidentiality of the information, but it can be made available upon reasonable request through the Corresponding author.
